# Therapeutic Potential of *Neopyropia yezoensis*: An Updated Review

**DOI:** 10.3390/md23110415

**Published:** 2025-10-23

**Authors:** Anshul Sharma, Na Young Yoon, Hae-Jeung Lee

**Affiliations:** 1 Department of Food and Nutrition, College of Bionanotechnology, Gachon University, Seongnam-si 13120, Republic of Korea; anshulsharma@gachon.ac.kr; 2 Institute for Aging and Clinical Nutrition Research, Gachon University, Seongnam-si 13120, Republic of Korea; 3 Food Safety and Processing Research Division, National Institute of Fisheries Science, Busan 46083, Republic of Korea; dbssud@korea.kr; 4 Department of Health Sciences and Technology, Gachon Advanced Institute for Health Science and Technology, Gachon University, Incheon 21999, Republic of Korea

**Keywords:** antioxidant, anti-inflammatory, anti-aging, seaweed, myosporin-like amino acids, neuroprotection

## Abstract

*Neopyropia* (*N.*) *yezoensis* is a widely cultivated red alga in East Asia and valued worldwide for its rich bioactive constituents recognized for their health benefits, including polsaccharides, porphyrans, pigments, phenolic compounds, phycobiliproteins, polyunsaturated fatty acids, myosporin-like amino acids, and both synthetic and recombinant peptides. This review summarizes the current knowledge regarding the therapeutic potential of *N. yezoensis* extracts and their bioactive compounds. Based on in vitro, ex vitro, and in vivo experimental data (including those on *Drosophila melanogaster* larvae), this review comprehensively discusses its antioxidant, anti-inflammatory, neuroprotective, anti-atopic dermatitis, anti-colitis, anticancer, anti-aging, anti-atrophy, metabolic health-promoting effects, improving renal health, proliferating, anti-osteoarthritic, anti-allergic, antibacterial, and antivirus activities. The prebiotic effect of *N. yezoensis* porphyran through modulation of the gut microbiota was also investigated. Studies have indicated that protein hydrolysates and peptides derived from *N. yezoensis* with low molecular weights and aromatic and/or hydrophobic amino acids contribute significantly to these diverse bioactivities. Although *N. yezoensis* has shown promising bioactivity in preclinical models, validated clinical data in humans are currently lacking. Future research should prioritize the design and implementation of well-controlled human clinical trials to fully explore their therapeutic potential.

## 1. Introduction

*Neopyropia* (*N.*) *yezoensis* (formally *Pyropia yezoensis*), an important medicinal seaweed, is a red macroalga (Rhodophyta) belonging to the order Bangiales and class Bangiophyceae. This species was previously known as *Porphyra yezoensis* [1]. In 2020, Brodie and Yang reclassified *Neopyropia* as a new genus within the Bangiales, based on molecular phylogenetic analyses that distinguished it from *Pyropia* and other related genera [2]. Species of *Pyropia* belonging to the order Bangiales include *P. columbina*, *P. crassa*, and *P. haitanensis*, while *Neopyropia* includes species such as *N. yezoensis*, *N. tenera*, and *N. leucosticta* [3]. It is commercially cultivated and consumed in various civilizations, including Korea, China, and Japan, where it is commonly famous as gim (김), zǐcài (紫菜), and nori (のり), respectively [4]. *N. yezoensis* is an important and popular Korean seafood that is used to roll rice and other ingredients in the traditional Korean dish gimbap [5]. Nori is an edible product in Japan that is consumed with sushi and green tea (cha), and hoshi-nori, toasted nori, and seasoned nori are common nori products in Japan [6]. Nori aquaculture commenced three centuries ago, and akin to other East Asian nations, susabi-nori (*N. yezoensis*) is currently the most appreciated seaweed crop in Japan [7]. It is commonly known as “laver” in Canada, the United Kingdom, and the United States of America, and “Karengo” in New Zealand [8]. 

Laver is a general term used to describe the different species of *Porphyra*, *Pyropia*, and *Neopyropia*. For example, the main laver species produced in Korea include *Neopyropia tenera* (formerly *Porphyra tenera*) (Kjellman) (Kikuchi et al., 2011), *Neopyropia yezoensis* (Ueda) Hwang and Choi, 2011; *Neoporphyra seriata* (Kjellman) Kikuchi and Miyata, 2011; and *Neoporphyra dentata* (Kjellman) (Kikuchi and Miyata, 2011). In South Korea, 20 different types of laver are cultivated, of which *N. yezoensis* and *N. dentata* are the predominant species [9,10]. Furthermore, in July 2017, the Codex Alimentarius and South Korea’s standards for laver products were recognized as standards in the Asian region, leading to the globalization of laver products [11]. Commercial laver species in China (*Pyropia haitanensis* (Chang and Zheng) Kikuchi and Miyata, 2011, *N. yezoensis*) and Japan (*N. yezoensis*, *N. tenera*, *Pyropia pseudolinearis* (Ueda) Kikuchi et al., 2011) have also been documented [12].

Korean laver accounts for more than 70% of the total laver production worldwide. Lavers are red seaweed species (*Porphyra*, *Pyropia*, and *Neopyropia* genera) that are mostly utilized as raw or processed food items (e.g., roasted, dried, or seasoned) or as suppliers of compounds with health benefits [13]. Furthermore, laver is a rich source of amino acids, including aspartic acid, betaine, glutamic acid, and taurine, and has a very low lipid content [14]. In a review, the nutritional values (proximate values, W/W%) of raw wet laver—carbohydrate (1.2–2.7), dietary fiber (-), protein (3.0–5.0), lipid (0.5), ash (3.6–4.3), and moisture (89.2–90.5)—and mineral and raw laver (dry weight)—carbohydrate (51.2–57.9), dietary fiber (-), protein (36.2–39.2), lipid (2.3–3.1), ash (3.8–7.3), and moisture (-), mineral, and amino acids, as well as processed laver—carbohydrate (45.4–50.0), dietary fiber (-), protein (29.3–35.0), lipid (1.8–2.0), ash (8.1–9.9), and moisture (8.2–9.8), along with mineral—have been documented [13]. The global laver trade has expanded at an average annual growth rate of 10.5%, from 440 billion USD in 2014 to 1.08 trillion USD in 2023 [15]. 

An earlier review discussed the important bioactive components of different species of *Pyropia* and their pharmacological effects, including their antioxidant, immunomodulatory, antihypertensive, anticancer, and anticoagulant properties, with some studies focusing on *N. yezoensis* [8]. A separate review outlined the health benefits of peptides derived from *N. yezoensis* [16]. Moreover, a recent review provided a detailed description of the extraction techniques, structural characteristics, and pharmacological activities of polysaccharides derived from *N. yezoensis*, including antioxidant activity, kidney stone suppression, anti-inflammatory activity, immuno-modulatory activity, hypolipidemic effects, and anti-osteoclastogenesis activity [17]. The current review presents an updated overview of the health-promoting properties of extracts, proteins, peptides, synthetic and recombinant peptides, polysaccharides, porphyran, oligo-porphyran, mycosporine-like amino acids, and fatty acids derived from *N. yezoensis*. This review presents previously unaddressed health benefits of *N. yezoensis* and uses an updated genus name *Neopyropia* for all previously published studies with old nomenclature.

## 2. Important Metabolites

*Neopyropia* has been recognized for its numerous pharmacological benefits that are attributed to a variety of nutrients and bioactive compounds, such as polysaccharides, porphyrans (sulfated polysaccharides), proteins, fatty acids, vitamins, minerals, and carotenoids [18]. Many critical factors influence the activity of polysaccharides, including the molecular weight, type of sugar, glycosidic branching, position of sulfate groups, and degree of sulfation [8]. Porphyran, a primary constituent of *N. yezoensis*, is a sulfated polysaccharide (linear) composed of repeating units of galactose and 3,6-anhydrogalactose. These units are connected by alternating alpha-1,4 and beta-1,3 glycosidic bonds. Oligo-porphyran is produced through the enzymatic breakdown of porphyran by porphyranase, resulting in shorter chains of galactose and 3,6-anhydrogalactose, which exhibit enhanced bioactivity and efficacy compared with porphyran [19].

The protein content of *N. yezoensis* was reported to be approximately 35% [20]. The high protein content of *N. yezoensis* makes it a potential source of bioactive peptides with significant health benefits. Bioactive peptides consist of short sequences ranging from three to 20 amino acids that are inactive within the parent protein [8]. The activity of bioactive peptides depends upon their amino acid sequence and composition [16,21]. Recombinant proteins have generated significant interest as therapeutic agents, demonstrating considerable potential for use in various human conditions including chronic diseases, infections, and cancer. The purification of algal proteins is characterized by high costs, significant time investment, and the need for a substantial quantity of parental algae. Researchers have used genetic engineering methods to express proteins in bacteria and evaluated them for therapeutic applications [22].

**Figure 1:**
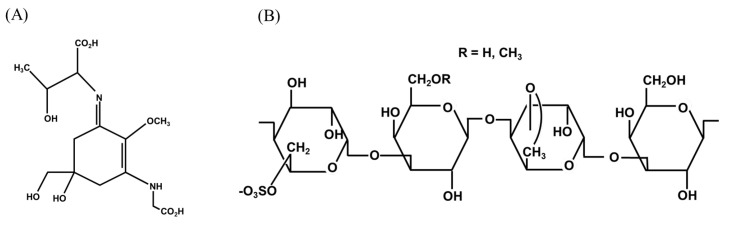
Structures of (**A**) MAA (**B**) porphyran. MAA: myosporin-like amino acid.

Many organisms employ strategies to protect themselves from oxidative stress and ultraviolet (UV) radiation damage. *N. yezoensis* synthesizes secondary metabolites called myosporin-like amino acids (MAAs) that function as effective antioxidants and protect against harmful UV radiation. Furthermore, the natural pigments present in *N. yezoensis* are classified as phycobiliproteins, including phycoerythrin, phycocyanin, and allophycocyanin. Phycoerythrin is categorized into “B,” “C,” and “R” types. R-phycoerythrin is the most prevalent phycobiliprotein with established biological activity [23]. Figure 1 shows the structure of porphyran and MAA [24]. 

Dai et al. [25] reported the proximate polysaccharide and protein-rich composition of *N. yezoensis* extracts from six different production areas in South Korea, Jinhae, Haenam, Jangheung, Jindo, Wando, and Sinan. The yield of extracts ranged from 10.20 ± 0.10 to 20.00 ± 0.20%. The proximate composition comprises polyphenol (1.31 ± 0.21 to 6.31 ± 0.21%), carbohydrate (23.28 ± 2.88 to 29.67 ± 2.90%), protein (20.78 ± 3.67 to 28.33 ± 3.67%), total sterols (0.21 ± 0.01 to 0.34 ± 0.01%), lipid (2.95 ± 0.01 to 4.06 ± 0.01%), ash (derived from the original sample, 10.00 ± 0.01 to 14.40 ± 0.01%), and moisture (derived from the original sample 5.04 ± 0.10 to 5.85 ± 0.10%). This compositional variance may be attributed to sample conditions, water temperature, and sunshine intensity [25].

Another study evaluated the nutritional, amino acid, and fatty acid profiles of the head water from two varieties of *N. yezoensis*, namely Sutong No.1 and Jianghaida No. 1. [26]. The nutrient composition (%, dry based) of the two varieties was as follows: crude protein (33.71 ± 0.31, 41.79 ± 0.23), total sugar (32.30 ± 0.50, 29.33 ± 1.07), crude fiber (1.93 ± 0.06, 3.13 ± 0.15), ash (10.24 ± 0.03, 4.28 ± 0.13), and crude fat (0.53 ± 0.01, 0.30 ± 0.03), respectively. Both varieties comprise 16 amino acids, with alanine exhibiting the highest amount and histidine the lowest. The mineral composition included 12 mineral elements, including sodium, magnesium, potassium, calcium, manganese, iron, zinc, phosphorus, selenium, copper, cadmium, and arsenic. The fatty acid composition, comprising 24 identified fatty acids, is presented in Table 1 [26]. The variety Jianghaida No. 1. was observed to be nutrient-rich among the two. Lee et al. [27] reported the chemical composition of a liposoluble fraction of *N. yezoensis* extract and identified total lipids, total fatty acids, ꞵ-carotene, and total sterols. Table 1 shows the fatty acid composition of *N. yezoensis* and comprises unsaturated and saturated fatty acids [27]. Further research is needed to assess the full pharmacological potential associated with this fatty acid composition.

## 3. Literature Search Strategy

In 2024, non-systematic bibliographic research was conducted using electronic web-based databases (Web of Science, Google Scholar, Embase, and PubMed). The following key words were used: “*Neopyropia* AND therapeutic effects,” “Laver/ nori AND therapeutic effects,” “*Neopyropia yezoensis* AND health-promotion,” *Neopyropia yezoensis* AND individual therapeutic effects, including “antioxidant,” “anti-inflammatory,” “antitumor,” “anti-ageing/skin protection,” “hepatoprotective,” “neuroprotection,” “anti-metabolic disorder,” “anti-atopic dermatitis,” “lipid lowering,” “renal protection,” “cardiovascular protection,” “anti-muscle atrophy” and “antibacterial.” Only the articles written in English were included in this review.

## 4. Therapeutic Effects

**Figure 2:**
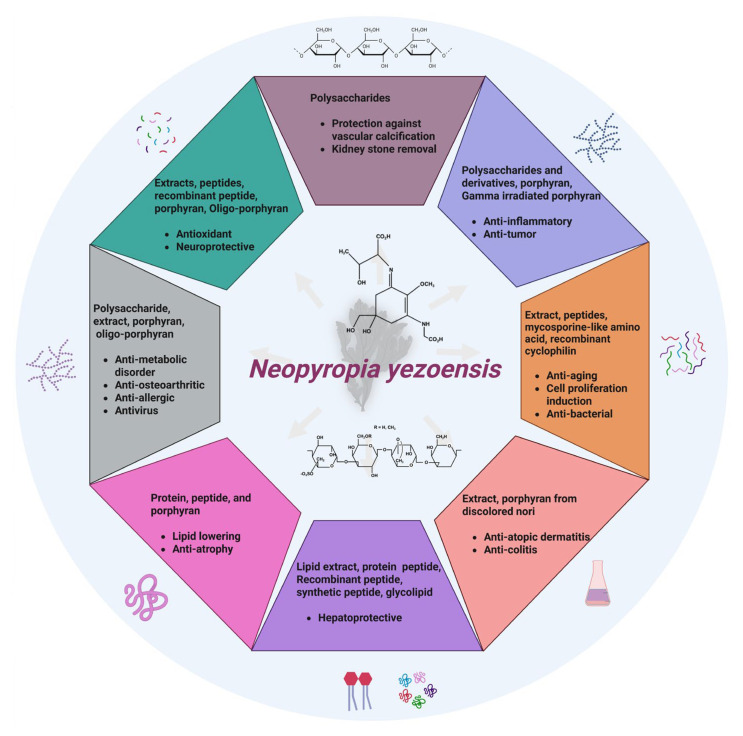
Health-promoting activities of *N. yezoensis.* Created using BioRender.

The following section comprehensively examines the wide range of health-promoting activities attributed to *N. yezoensis* (Figure 2).

### 4.1. Antioxidant

*N. yezoensis* is an intertidal alga, and numerous studies have recorded its tolerance to biotic and abiotic stresses [28,29,30]. Polyphenols and proteins are also potent antioxidants. Dai et al. [25] reported the antioxidant effects of *N. yezoensis* extracts sourced from six different locations of South Korea, both in vitro and in vivo. The extracts were rich in polyphenols and proteins. The extracts demonstrated radical-scavenging activity against 1,1-diphenyl-2-picryl-hydrazyl and alkyl free radicals. Moreover, the extracts removed reactive oxygen species (ROS) from Vero (mammalian kidney) cells subjected to oxidative stress generated by 2, 2′-azobis (2-amidinopropane) dihydrochloride. Furthermore, the Jindo and Sinan exhibited protective effects against apoptosis. In zebrafish, the extract reduced ROS levels, lipid peroxidation, and cell damage. This study showed that regardless of regional influence, *N. yezoensis* extract possesses potential antioxidant agents both in vitro and in vivo [25] (Table 2).

In an interesting study, Kim et al. [31] identified and synthesized phycobiliproteins from *N. yezoensis* and assessed their antioxidant activity in H_2_O_2_-induced human hepatocellular carcinoma (HepG2) cells. Of the 13 peptides, PBP2 (0.01, 0.1, and 1 μg/mL) exhibited good efficacy in suppressing ROS generation, enhancing cell survival, and averting cell death in comparison to the ascorbic acid control (1 µg/mL). Moreover, regulation of the nuclear factor erythroid-derived 2-like 2 (Nrf2)/superoxide dismutase (SOD) pathway by PBP2 alleviates H_2_O_2_-stimulated stress. This study presents the antioxidant activity of peptides involved in the Nrf2/SOD signaling pathway [31]. 

Similarly, the antioxidant activities of peptides prepared using various enzymatic digestions of *N. yezoensis* demonstrated their radical-scavenging properties and protective effects against HepG2 cells stimulated with N-acetyl-p-aminophenol (APAP, acetaminophen). The enhanced antioxidant activity of protein hydrolysates could be due to the release of compounds that promote the antioxidant activity compared to the unhydrolyzed protein. The antioxidant activity was dose-dependent. This study indicates that protein hydrolysates may serve as viable substitutes for synthetic antioxidants [32] (Table 2). 

A separate study reported the enhanced antioxidant activity in *N. yezoensis* fermented with *Bacillus amyloliquefaciens* MMB-02, *Lactiplantibacillus plantarum* L13, and *Saccharomyces cerevisiae* A8, compared to the untreated control. LC-MS/MS analysis revealed the presence of hydrophobic residues of amino acids and oligopeptides that may have contributed to the antioxidant properties which may have originated mainly from phycocyanin and phycobiliproteins [33]. 

Gold 1 (G1), a strain of *N. yezoensis* resistant to red rot disease, was recently compared to a commercial *N. yezoensis* strain (CP) with antioxidant activity. The aqueous extract of G1 exhibited greater 2,2′-azino-bis (3-ethylbenzothiazoline-6-sulfonic acid) scavenging activity than the commercial strain. Moreover, G1 treatment mitigated H_2_O_2_-induced human embryonic kidney (293T) cell death in a dose-dependent manner, with the highest concentration yielding results comparable to those of the positive control, quercetin. The cytoprotective effect of CP was weaker than that of G1. The ROS-mitigating effect was exclusively observed for G1 at all concentrations, whereas CP exhibited no effect. These results indicated that G1 diminished cell death by reducing ROS levels in H_2_O_2_-treated cells. The study also assessed the anti-apoptotic effect of G1, as indicated by alterations in the expressions of several apoptosis-related genes. Overall, the antioxidant and anti-apoptotic effects of G1 surpassed those of the commercial strain [34]. Future studies should assess the efficacy of this strain in combating oxidative stress-induced diseases using diverse cell and animal models. 

Immunophilins are a highly conserved family of proteins comprising two main members: cyclophilins and FK506-binding proteins. They have the capacity to bind to immunosuppressive drugs [37]. Cyclophilins exhibit peptidyl–prolyl isomerase (PPIase) activity and have been implicated in antioxidant activity and regulation of immune responses. Kim et al. [35] first assessed the antioxidant activity of a recombinant cyclophilin-type peptidylprolyl cis-trans isomerase isolated from *N. yezoensis* against HepG2 cells induced using H_2_O_2_. Recombinant proteins exhibit antioxidant properties by decreasing ROS production, increasing cell viability, and inhibiting oxidative damage and mitochondrial malfunction. This protein restores the expressions and activity of SOD, CAT, thioredoxin reductase (TRR) and glutathione peroxidase (GPx) in HepG2 cells [35]. Recently, *N. yezoensis* polysaccharides were reported to demonstrate comparable or superior radical scavenging activity compared to other natural polysaccharides (*Brasenia schreberi* water-soluble polysaccharides and litchi polysaccharides). Although the scavenging potential was observed to be lower than the control (vitamin C). The functional groups of polysaccharides can freely interact with free radicals to form non-covalent or hydrogen bonds [36]. Future studies should investigate the diverse antioxidant signaling mechanisms underlying the antioxidant activity of this recombinant protein. Overall, the results of these studies indicate the potential of the various constituents of *N. yezoensis* as antioxidants. In the future, cell and animal-based studies are required.

### 4.2. Anti-Inflammatory

*N. yezoensis* extracts have been investigated for various medicinal applications, particularly for their potential anti-inflammatory properties. Consequently, the search for potential anti-inflammatory agents has received increasing attention. Table 3 illustrates in vitro studies that revealed the anti-inflammatory activities of the bioactive components of *N. yezoensis*.

Porphyrans are indigestible dietary fibers found in *N. yezoensis* that exhibit many health-promoting properties. A previous study reported that porphyran extracted from *N. yezoensis* suppresses nitric oxide (NO) generation in lipopolysaccharide (LPS)-stimulated macrophages (RAW264.7 cells) dose-dependently by inhibiting NF-κB activation, suggesting an anti-inflammatory effect of porphyran [38]. In further research, the team prepared porphyrans from discolored nori and evaluated their anti-inflammatory properties using an in vitro model. Discolored nori has no commercial value, as it is devoid of nutritional value owing to deficiencies in nitrogen (N), phosphorus (P), and trace elements during its cultivation [42]. This study indicates that the molecular size significantly influences the anti-inflammatory activity of porphyrans in LPS-stimulated RAW264.7 cells. Specifically, D2-porphyran, characterized by the smallest molecular size fraction as determined by size-exclusion chromatography, demonstrated the highest inhibitory activity against NO and tumor necrosis factor-alpha (TNF-α) secretion in comparison to other porphyrans. The combined effect of hydrogen peroxide and ascorbate on free radical degradation significantly enhanced the inhibitory activity of the four porphyrans. Following degradation, porphyran D2 exhibited increased capacity to repress the receptor activator of NF-κB ligand-induced osteoclastogenesis in RAW264.7 cells [39]. 

Previous research has indicated that waste-discolored nori serves as a good source of porphyrans, exhibiting superior antioxidant and anti-inflammatory activities relative to porphyrans derived from standard nori [42]. Porphyran can play a dual role as a stimulator and an inhibitor because of its interaction with several binding proteins. A previous study investigated the immuno-suppressive effects of porphyran on LPS-induced human immune cells, specifically peripheral blood dendritic cells, peripheral blood mononuclear cells, and monocyte-derived dendritic cells. The suppressive mechanism of porphyran arises from its ability to compete with LPS for binding to myeloid differentiation factor 2, thereby suppressing immune cells [40]. Another study demonstrated the protective effect of porphyran against LPS-triggered immune activation, which was facilitated by the competitive binding of porphyran with LPS in mouse spleen dendritic cells [41]. The results of this study suggest a promising therapeutic role for porphyrans. Future studies should prioritize the use of animal models.

### 4.3. Neuroprotective

The endoplasmic reticulum (ER) regulates cellular calcium levels and modifies and cross-links proteins. Cellular stress-triggered ER stress responses have been frequently examined to elucidate the neuroprotective effects of these compounds. Table 4 shows the neuroprotective effects of *N. yezoensis*.

Oh et al. [43] conducted a study using phycoerythrin (PYP) extracted from the tryptic peptide of *N. yezoensis* to assess its neuroprotective effects on rat primary hippocampal neurons, specifically by reducing glutamate-induced ER stress. The research elucidated that PYP alleviates glutamate excitotoxicity by suppressing the glucose-regulated protein 78 (GRP78, an indicator of ER stress) and inhibiting c-Jun N-terminal kinase (JNK) phosphorylation. Moreover, it decreased the activity of senescence-associated β-galactosidase (SA-β-Gal) in aged hippocampal neuron cells (primary) and mitigated the age-related degeneration of neurites. This study demonstrated that PYP regulates ER stress through the activation of tropomyosin-related kinase B (TrkB)-phosphatidylinositol 3-kinase (PI3K)-extracellular signal-regulated kinase 1/2 (ERK1/2) signaling, which prevents glutamate-induced excitotoxicity. Moreover, PYP reduced hippocampal neuronal senescence by relieving age-dependent ER stress and neurite degeneration [43]. 

Additionally, the protective effects of PYP have been reported in perfluorooctane sulfonate (PFOS)-stimulated rat frontal cortical neurons. Research has indicated that the administration of phycoerythrin peptide promotes the survival of frontal cortical neurons by inhibiting PI3K-ERK1/2 activation, obstructing the TrkB receptor, and attenuating ER stress triggered by PFOS exposure in the rat prefrontal cortex. PYP treatment lowered the increase in intracellular calcium levels and phosphorylation of c-Jun N-terminal kinase and calcium/calmodulin-dependent protein kinase II, which are related to a PFOS-triggered increase in GRP78. Both studies revealed that peptides derived from *N. yezoensis* prevented neuronal damage by lowering ER stress [44].

Another study outlined the neuroprotective role of an oligo-porphyran derived from the porphyrin of *N. yezoensis*. In 6-hydroxydopamine hydrochloride-induced rat pheochromocytoma (PC12) neuronal cells, oligo-porphyran attenuates neurotoxicity by reducing ROS production and lactate dehydrogenase release. Oligo-porphyran primarily inhibited the mitochondria-mediated apoptotic pathway and upregulated the PI3K/Akt pathway. These studies indicated that compounds extracted from *N. yezoensis* possess neuroprotective properties [45]. These studies suggest the potential role of various bioactive components derived from *N. yezoensis* as neuroprotective agents. Subsequent investigations are required to concentrate on the effects observed in animal models.

### 4.4. Anticancer

Cancer represents a major global health challenge and is devoid of effective and comprehensive treatments. Consequently, alternative therapies incorporating natural ingredients have been proposed. The extraction and assessment of marine components as anticancer agents have received considerable attention from the scientific community. The antitumor properties of seaweeds may result from the presence of diverse bioactive constituents and nutrients [46]. Studies have documented the anticancer properties of various porphyran and polysaccharide fractions derived from *N. yezoensis* (Table 5).

Gamma radiation treatment of porphyran from *N. yezoensis* Chonsoo2 (PYP) resulted in the formation of two fragments, specifically the PYP-20 and PYP-50 derivatives. Three porphyrans exhibited antitumor activity against the human cervical cancer cells (HeLa), the human hepatic carcinoma cells (Hep3B), and the human breast carcinoma cells (MDA-MB-231). Research indicated that the high molecular weight fraction of porphyran shows superior antiproliferative activity against HeLa cells compared to the low molecular weight fractions. PYP demonstrated a greater inhibition ratio in HeLa cells than 5-fluorouracil and its derivatives. This indicates that the molecular weight and conformation of porphyran may influence its biological properties [47].

In contrast, low-molecular-weight derivatives generated by gamma irradiation of polysaccharides isolated from *N. yezoensis* Sookwawon 104 (PYSP) demonstrated greater efficacy in inhibiting the cell cycle in Hep3B, HeLa, and MDA-MB-231 cells. The fractions PYSP-20 and PYSP-100 effectively inhibited tumor cells by regulating expressions of P21, P53, Cyclin B1, and Cdk1. The superior activity of the derivatives may be attributed to the variation in molecular weight compared to that of PYSP. These studies suggest that, in addition to molecular weight, the conformation and strain type of seaweeds may influence antitumor activity [48]. 

Galactan, a sulfate polysaccharide (GPY_crude_) along with its derivatives (GPY_10_ and GPY_300_), was tested by Pham et al. [49] for anticancer activity in human prostate cancer cells (PC-3 and DU145). The obtained fractions exhibited the same monosaccharide compositions yet varied in molecular weights. GPY_10_ demonstrated the highest prebiotic activity and showed the greatest inhibition of DU145 cells compared to the other two galactans. GPY_10_ stimulates ROS production, induces apoptosis in DU145 cells and targets the PI3K/AKT/mTOR signaling pathway. This study suggests that low-molecular-weight galactan is ideal for anticancer activity against prostate tumor cells. Future studies are required to evaluate these effects in vivo [49]. 

### 4.5. Anti-Atopic Dermatitis

Atopic dermatitis (AD) is a chronic inflammatory disease triggered by interferon (IFN)-γ and TNF through the activation of signaling pathways, leading to the production of proinflammatory molecules. Suppression of mitogen-activated protein kinases (MAPKs) decreases the synthesis of proinflammatory cytokines and associated intracellular signaling pathways, thereby preventing the activation of NF-κB [50]. Researchers have evaluated the anti-AD effects of *N. yezoensis* (Table 6). 

**Figure 3:**
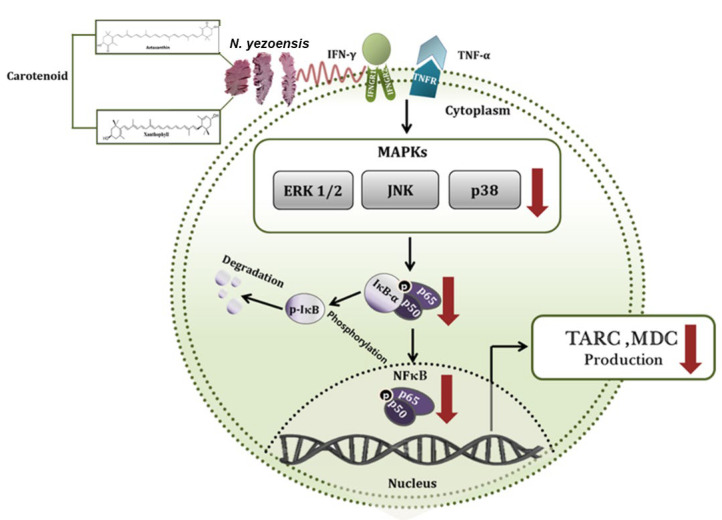
Schematic inhibitory signaling pathway of *N. yezoensis* extract (PYE) on interferon (IFN)-γ- and tumor necrosis factor (TNF)-α-induced TARC and MDC production in human keratinocytes. Red poly line arrows indicate the activity of *N. yezoensis* extract (PYE) and black arrows indicate the induction of cytokines. Adapted from MDPI with kind permission from [51] with little modifications. TARC: thymus and activation-regulated chemokine; MDC: macrophage-derived chemokine.

Ha et al. [51] reported that the extract of *N. yezoensis* reduced the generation of inflammatory-mediated chemokines (macrophage-derived chemokine, thymus and activation-regulated chemokine) in HaCaT (immortalized human keratinocytes) cells triggered by interferon (IFN)-γ/TNF-α. This effect was attributed to the downregulation of NF-κB and MAPK pathways (Figure 3). Moreover, high-pressure liquid chromatography (HPLC) analysis identified two carotenoids, astaxanthin and xanthophyll, as potential anti-inflammatory compounds in the tested extract. The results showed that *N. yezoensis* may alleviate AD [51] (Table 6). 

### 4.6. Anti-Colitis

Ulcerative colitis is a subtype of inflammatory bowel disease characterized by inflammation of the mucosal (innermost lining) layer, with limited therapeutic options. The condition appears to be multifactorial, associated with genetic factors, dysregulated immune responses, defects in the epithelial barrier, gut microbiota, and environmental factors [52]. Colitis results from the intrusion and damage of colon villi by immune cells, hindering the ability of the colon to absorb water, and leading to diarrhea characterized by loose, watery, and frequent bowel movements. Various types of immune cells, such as dendritic cells, macrophages, natural killer cells, and T cells, play crucial roles in the pathophysiology of the disease.

**Table 6:** The anti-atopic dermatitis and anti-colitis effects of *N. yezoensis*.

Test Material	Experimental Model	Outcomes/Mechanisms	Ref.
Anti-atopic dermatitis			
*N. yezoensis* extract (PYE)	HaCaT Cells+ PYE (40, 200, and 1000 µg/mL), TNF-α or IFN-γ (10 ng/mL), 24 h	↓ TARC, ↓ MDC, ↓ ERK, ↓ JNK, ↓ p38, ↓ NF-κB	[51]
Anti-colitis			
Porphyran from decolored *N. yezoensis*	C57BL/6 mice + DSS (4%, 8 days), replaced every two days + porphyran (50 mg/kg, 100 μL) orally or intraperitoneal, 7 days	Reversed BW reduction, ↓ DAI score, ↓ colon length, ↓ inflammatory cell infiltration. In colon: ↓ CD11c, ↓ DCs, ↓ TCR-β+, ↓ NK, ↓ neutrophils. ↓ CD11c (macrophages). In mesenteric lymph nodes CD4 T cells: ↓ IFN-γ, ↓ IL-17 CD8 T cells: ↓ IFN-γ, ↓ IL-17, ↓ T-bet, ↓ RORγt Serum: ↓ IFN-γ, ↓ IL-17. Colonic DCs and macrophages: ↓ CD40, ↓ CD80, ↓ CD86. ↓ IL-1β, ↓ IL-12, ↓ IL-6, ↓ IL-23	[53]

BW: body weight; CD: cluster of differentiation; DAI: disease activity index; DCs: dendritic cells; DSS: dextran sulfate sodium salt; ERK: extracellular signal-regulated kinase; HaCaT: human keratinocyte cells; IFN-γ: interferon; IL: interleukin; JNK: c-Jun N-terminal kinase; MDC: macrophage-derived chemokine; NF-κB: Nuclear Factor kappa B; NK: natural killer; p38 MAPK-mitogen-activated protein kinase; RORγt: RAR-related orphan receptor-γ; TARC: thymus and activation-regulated chemokine; T-bet: transcriptional factor; TCR-β+: T cell receptor-β+; TNF-α: tumor necrosis factor-alpha. ↑ = increases and ↓ = decreases.

A recent study from Korea reported the ameliorating effects of porphyran on dextran sodium sulfate-induced acute and chronic colitis in vivo. Porphyran treatment suppressed dendritic cells and macrophage activation, as evidenced by a significant reduction in the levels of co-stimulatory molecules, specifically cluster of differentiation (CD)40, CD80, and CD86. The levels of co-stimulatory receptors on macrophages were also diminished. Porphyran blocks pathogen- and damage-associated molecular pattern-dependent activation of dendritic cells and macrophages. Porphyran did not promote the activation or differentiation of T cells (Table 6). Porphyran supplementation also reduced chronic colitis. This study suggests that porphyran is a viable natural alternative for the treatment of both acute and chronic colitis [53].

### 4.7. Anti-Aging

Skin aging is triggered by extrinsic factors such as UV radiation and environmental pollutants, as well as intrinsic factors, including reduced levels of extracellular matrix components such as collagen and elastin. Mycosporine-like amino acids (MAAs) remarkably safeguard against photo-induced skin aging. Ryu et al. [54] evaluated the protective effects of MAA from *N. yezoensis* against extrinsic aging induced by UV irradiation in human skin fibroblasts (CCD-986sk). HPLC and electrospray ionization mass spectrometry identified porphyra-334, palythine, and asterina-330 MAAs in *N. yezoensis*. After UVA irradiation, the dominant fraction, porphyra-334, suppressed the production of ROS and the expression of MMPs, while enhancing the levels of procollagen, elastin, and type I collagen. This study suggests the potential protective effects of porphyra-334 against photoaging. However, further investigations are needed to elucidate the underlying signaling mechanisms [54] (Table 7). 

Type I collagen accounts for approximately 85% of the total collagen and is degraded by matrix metalloproteinases (MMPs). On the other hand, the enzyme tissue inhibitor of metalloproteinases (TIMPs) inhibits the activity of MMPs. Collagen synthesis is associated with multiple signaling pathways. To identify a plausible pathway, one study reported the effect of a peptide (PYP1–5) from *N. yezoensis* on collagen synthesis using an in vitro human dermal fibroblast cell line (Hs27). Treatment with PYP1–5 enhances the expression of type I collagen. The peptide increased the expression of TIMP-1 and TIMP-2, while reducing the expression of MMP-1 at the mRNA and protein levels. The study indicated the activation of the transforming growth factor-β (TGF-β)/Smad signaling pathway in collagen synthesis by the peptide. This study suggested a protective effect of PYP1–5 against intrinsic aging [55] (Table 7). Further studies are required to assess the anti-aging potential of the bioactive constituents of this seaweed.

In contrast to collagen synthesis, melanogenesis contributes to hyperpigmentation and aging. Melanin production is affected by UV radiation and hormonal changes. A recent study evaluated the efficacy of *N. yezoensis* extract as *a* preventive agent against skin pigmentation and aging in various skin cell lines, including human dermal keratinocytes (HaCaT), human dermal fibroblasts (1064 SK), and mouse melanocytes (Melan-A). The extract inhibited melanogenesis, collagen-degrading enzymes, and tyrosinase activity while promoting enzymes associated with procollagen synthesis. The decrease in *N. yezoensis* tyrosinase activity was less pronounced than that observed in the positive control (arbutin). Moreover, the clinical evaluation of *N. yezoensis* extract applied as a simple lotion in 23 female volunteers showed enhanced skin brightening and reduced melanin content (Table 7). This finding shows the potential of *N. yezoensis* extract as an effective cosmetic and anti-aging agent [56]. 

**Figure 4:**
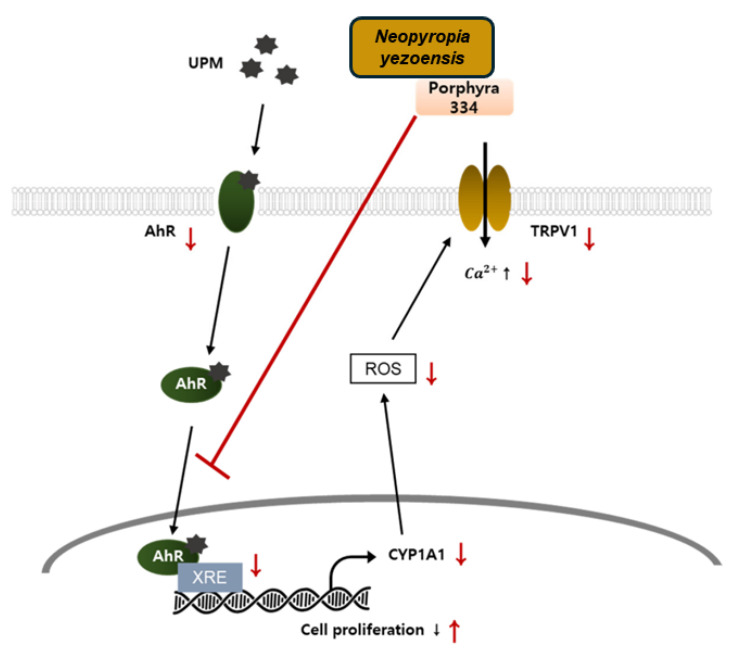
Action mechanism of porphyra 334 in the AhR and TRPV1-mediated signaling. Porphyra 334 inhibits UPM effects by inhibiting both AhR and TRPV1 signaling. Red line: action step of porphyra 334. A red arrow pointing up indicates activation, and a red arrow pointing down indicates inhibition (adapted from MDPI with kind permission from [57] with little modifications). AhR: aryl hydrocarbon receptor; ROS: reactive oxygen species; TRPV1: transient receptor potential vanilloid 1; UPM: urban particulate matter.

In a recent study, *N. yezoensis* water extract and Porphyra 334 (its active metabolite) ameliorated keratinocyte damage triggered by urban particulate matter (UPM) by suppressing aryl hydrocarbon receptor-induced ROS generation, which reduced the activity of transient receptor potential vanilloid 1, thereby allowing the proliferation of keratinocytes (Figure 4). This study emphasized the importance of Porphyra 334 in the context of skin diseases, indicating its anti-pollutant activity [57]. 

### 4.8. Induction of Cell Proliferation and Related Signaling Pathway 

Cell proliferation is essential for replenishing old and dead cells from body organs and sustaining the normal functioning of the body. The intestinal epithelium is acknowledged as one of the fast-growing tissues in the body [58]. The induction of cell proliferation is initiated by external signals that activate multiple intracellular signaling pathways, facilitating cell growth and proliferation. The influence of various natural products on the initiation of cell proliferation has been previously reported (Table 8). Researchers have focused on the bioactive components derived from seaweeds for the induction of cell proliferation. Receptor tyrosine kinases, such as insulin-like growth factor-I receptor (IGF-IR) and epidermal growth factor receptor (EGFR), are present on many cell surfaces and are crucial for signaling. Activation of IGF-IR and EGFR signaling plays key roles in cell survival and proliferation [59,60].

Lee et al. [61] investigated the effect of *N. yezoensis* peptide (PY-PE) on the proliferation of rat intestinal epithelial (IEC-6) cells and related signaling pathways. IGF-IR has been identified as an intracellular mechanism through which PY-PE promotes IEC-6 proliferation [61] (Table 8). In further research, the authors reported the role of the EGFR signaling pathway in the proliferation of IEC-6 cells, utilizing a peptide (PYP1 (1–20)) derived from *N. yezoensis*. These studies suggest the potential role of bioactive peptides from *N. yezoensis* as essential factors for intestinal cell proliferation [62]. Future studies are required to evaluate these effects on additional biological functions (Table 8). More recently, recombinant cyclophilin was synthesized from *N. yezoensis* cyclophilin protein and assessed for its activity in IEC-6 cells. Recombinant cyclophilin stimulates IEC-6 proliferation by activating the EGFR/renin-angiotensin system (Ras)/extracellular signal-regulated kinase (ERK) signaling pathway, and advances the cell cycle by regulating the G1/S phase through the upregulation of cyclins [63].

### 4.9. Anti-Atrophy

Skeletal muscle mass constitutes over 40% of body mass, and its reduction in mass and strength is termed muscle atrophy. Sarcopenia, characterized by muscle atrophy associated with aging, significantly affects the quality of life of the elderly [64]. Because of their established health benefits, proteins and peptides derived from *N. yezoensis* have been evaluated for their anti-atrophic effects in both in vitro and in vivo models. The authors elucidated the mechanisms related to signaling pathways that contribute to anti-atrophy effects (Table 9).

Numerous studies have evaluated the impact of the synthetic glucocorticoid dexamethasone as an atrophy-inducing agent. Dexamethasone-triggered muscle atrophy is facilitated by the stimulation of proteolytic systems, such as autophagy/lysosome, ubiquitin-proteasome, and calcium-dependent pathways [65]. Muscle atrophy involves the activation of genes associated with muscle degradation, including atrogin1/muscle atrophy F-box (MAFbx) and muscle RING finger protein 1 (MuRF1), as well as O-type forkhead transcription factors (FoxO1 and FoxO3a), and NF-κB. In addition, the roles of signaling pathways, including the insulin-like growth factor-I receptor (IGF-IR) and Akt-mTORC1 signaling pathways, were elucidated.

**Table 9:** Studies demonstrating the anti-atrophy effects of *N. yezoensis*.

Test Material	Experimental Model	Outcomes/Mechanisms	Ref.
Peptide (PYP1-5)	C2C12 myotubes + DEX (100 μM) + PYP1–5 (500 ng/mL), 24 h	↓ MAFbx, ↓ MuRF1	[66]
Peptide (PYP15)	C2C12 myotubes + DEX group (100 μM), DEX + PYP15 group (100 μM + 500 ng/mL), and PYP15 group (500 ng/mL)	↑ p-mTOR, ↑ p-IGF-IR, ↑ Raptor, ↑ p-Akt, ↑ p-IRS-1, ↑ REDD1, ↑ KLF-15, ↑ p-p70S6K, ↑ p-S6, ↑ p-4E-BP1, ↑ eIF4E, ↑ p-FoxO1, ↑ p-FoxO3a, ↓ 20S proteasome activity. Downregulation of autophagy lysosomal system	[67]
Crude protein (PYCP)	C2C12 myotubes + DEX exposure (100 μM) + PYCP (20 and 40 µg/mL), 24 h	Dose-dependent increase myotube diameter, ↑ myogenin, ↑ p-FoxO1, ↑ p-FoxO3a, ↓ MAFbx, ↓ MuRF1, ↓ 20S proteasome activity, ↓ cathepsin-L, ↓ LC-I to LC-II	[68]
Protein (PYCP)	C57BL/6 mice + DEX (3 mg/kg BW, I.P.) + PYCP (oral administration, 150 and 300 mg/kg BW)	Prevented reduction in BW, calf thickness, ↑ gastrocnemius, ↑ tibialis anterior muscle weight, ↓ glucose levels, ↓ CK, ↓ LDH, In gastrocnemius muscle: ↑ p-Akt, ↑ p-IGF-IR, ↑ p-mTOR, ↑ Raptor, ↑ Rheb protein,↑ p-IRS-1, ↑ p-p70S6K, ↑ p-S6, ↑ p-4E-BP1, ↑ eIF4E	[69]
Protein (PYCP)	C2C12 myotubes + TNF-α (20 ng/mL) + PYCP (25, 50, and 100 µg/mL), 48 h	↑ myotube diameter, ↓ ROS, ↓ TNF-R1. Cytosolic: ↓ p-IκBα, ↑ NF-κB, Nuclear: ↓ NF-κB, ↓ atrogin-1/MAFbx, ↓ MuRF1, ↓ 20S proteasome activity, ↓ IL-6, ↑ MyoD, ↑ myogenin	[70]

BW: body weight; CK: creatine kinase; DEX: dexamethasone; eIF4E: eukaryotic translation initiation factor 4E; FoxO: forkhead box O; I.P.: intraperitoneal; KLF-15: Krüppel-like factor 15; LDH: lactate dehydrogenase; MAFbx: muscle atrophy F-box; MuRF1: muscle RING-finger protein-1; MyoD: myoblast determination protein 1; NF-κB: nuclear factor-κB; p-4E-BP1: phosphorylation of binding protein 1; p-Akt: phosphorylation of protein kinase B; p-IGF-IR: phosphorylation of insulin-like growth factor I receptor; p-IRS-1: phosphorylation of insulin receptor substrate 1; pIκBα: phosphorylated IκBα; p-mTOR: phosphorylation of mammalian target of rapamycin; REDD1: regulated in development and DNA damage response 1; ROS: reactive oxygen species; TNF-α: tumor necrosis factor-alpha; TNF-R1: TNF receptor-1. ↑ = increases and ↓ = decreases.

Mouse skeletal muscle cell (C2C12) myotubes serve as an in vitro model to study muscle atrophy. Lee et al. [66] found that PYP1-5, a peptide derived from *N. yezoensis*, inhibits the dexamethasone-induced muscle atrophy in C2C12 myotubes. Peptide treatment resulted in the downregulation of MAFbx and MuRF1 expression at both the transcriptional and translational levels, indicating protective effects [66]. In a persistent effort, peptide (PYP15) supplementation effectively blocked nuclear translocation of FoxO1 and FoxO3a by promoting their phosphorylation and inhibiting 20S proteasome activity. Moreover, the peptide regulated the associated IGF-I and Akt/mammalian target of rapamycin (mTOR)-forkhead box O (FoxO) signaling pathways in dexamethasone-induced C2C12 myotubes. PYP15 treatment inhibits proteolytic systems via Akt activation, thereby preventing muscle atrophy [67]. 

Although these peptides exhibit promising effects, they are hindered by disadvantages associated with their vulnerability to degradation and aggregation, primarily because of proteolytic enzymes found in the gastrointestinal tract. Previous studies evaluated the effects of *N. yezoensis* proteins on muscle atrophy. The protective effects of crude protein (PYCP) have been documented in dexamethasone-induced models of atrophy, both in cellular and animal studies. The crude protein extract (PYCP) significantly reduced the expressions of FoxO1 and FoxO3a. Moreover, PYCP suppresses the activation of the ubiquitin-proteasome and autophagy-lysosome pathways in dexamethasone-treated C2C12 myotubes [68]. 

In addition, oral administration of PYCP effectively mitigated the reduction in body weight, calf thickness, and muscle weight and decreased serum levels of creatine kinase (CK) and lactate dehydrogenase (LDH). LDH and CK are common serum markers of muscle damage. Moreover, PYCP regulates the IGF-I/Akt/rapamycin-sensitive mTOR complex I/FoxO signaling pathway. Inhibition of the autophagy/lysosome and ubiquitin-proteasome pathways has been identified as a mechanistic implication of PYCP [69].

Besides dexamethasone, the role of TNF-α has been implicated in muscle atrophy. Lee et al. [70] conducted perpetual research demonstrating PYCP exerts protective effects on TNF-α-induced C2C12 myotubes by suppressing the NF-κB signaling pathway through the elimination of intracellular ROS via its antioxidant activity [70] (Table 9). Future research should concentrate on investigating additional mediators of catabolic pathways, such as interleukin-6.

### 4.10. Metabolic Health-Promoting Effects

The lipid-lowering properties of *N. yezoensis* help to alleviate the effects of dyslipidemia, which can increase hepatic steatosis and damage. Their hepatoprotective properties protect the liver, the primary regulator of metabolic balance, facilitating effective lipid and glucose metabolism. These diverse effects work together to help with metabolic diseases, demonstrating the importance of *N. yezoensis* for restoring the body’s metabolic health. Among the bioactive components of *N. yezoensis,* porphyrans possess many health-promoting properties, including lipid-lowering effects. The hot-water extraction yield of porphyran from *N. yezoensis* (PPYP) was adjusted using response surface methodology, and its lipid-lowering effects were evaluated both in vitro and in vivo. This study revealed that porphyran (a sulfated hetero rhamno-galactan) significantly reduced triglyceride levels in palmitic acid-treated HepG2 cells and high-sucrose-fed *Drosophila melanogaster* larvae. The larval fat body is regarded as a functional homolog of human hepatic tissue. PPYP exerted lipid-lowering effects by reducing lipogenesis and enhancing fatty acid oxidation, and modulating the expression of lipid metabolism genes. Additionally, the regulation of numerous genes associated with lipid metabolism was observed [71] (Table 10).

Another study reported the hypolipidemic effects of porphyran extracted from *N. yezoensis* in an animal model subjected to a high-fat diet. Post 28-day porphyran treatment, there was a reduction in weight gain and an improvement in the serum lipid profile, comparable to the effects of a hypolipidemic drug (Zhibituo, approved in China). Moreover, porphyran treatment improved liver weight and ameliorated hepatic lipid composition [72] (Table 10). These studies suggest a potential lipid-lowering application of porphyran derived from *N. yezoensis*.

Many studies have reported the hepatoprotective properties of the extracts and bioactive components of *N. yezoensis*. Acetaminophen is a safe and effective fever-reducing and pain-relieving drug when administered at recommended doses. However, the incidence of acetaminophen toxicity is increasing owing to overdose, leading to serious hepatic and kidney injuries. Consequently, researchers are seeking safer alternatives [82]. 

Researchers have focused on marine components, and *N. yezoensis* has been identified as a potential hepatoprotective alternative (Table 10). The first report regarding the protective effects of *N. yezoensis* protein (PYP) extracted via hot water was in the context of acetaminophen-induced liver injury in Sprague–Dawley rats [73].

In continuous efforts, the researchers reported the characterization and protective activity of *N. yezoensis* protein (PYP) against acetaminophen-triggered cell mortality in Chang liver cells (CCL-13). The PYP portion consisted of two fractions, PYP1 and PYP2. The researchers synthesized the peptide PYP1, comprising 20 amino acid residues from the N-terminal of the PYP1 fraction, and documented its protective activity against acetaminophen-induced CCL-13 cells [74]. Moreover, the protective effects of recombinant peptides (expressed in *E. coli*) specified as PYP1, PYP1-AC, and PYP1-B, with molecular weights of 15, 12, and 5 kDa, respectively, along with a synthetic peptide, in the acetaminophen-triggered Chang liver cell line (HPV-18), have also been documented [75] (Table 10).

A previous report documented the protective effects of a synthetic peptide (PYP1–4) against acetaminophen-triggered damage in HepG2 cells. Treatment with PYP1–4 reduced ROS levels and upregulated expression of heme oxygenase 1 (HO-1), SOD2, catalase (CAT), and quinone oxidoreductase 1. Peptide treatment also decreased the growth inhibition and apoptosis of HepG2 cells. Moreover, the insulin-like growth factor 1 receptor signaling pathway suppresses apoptosis and necrosis [76]. Studies have indicated the protective effects of bioactive peptides in vitro, and subsequent research should assess their effects in animal models and across various signaling pathways.

Lipids derived from *N. yezoensis* have been reported to exhibit health-promoting properties, particularly hepatoprotective effects. Yanagita et al. [77] demonstrated the ameliorating effects of eicosapentaenoic acid (EPA)-containing polar lipids from *N. yezoensis* against obesity-induced hepatic steatosis, hepatic injury, and hepatomegaly in obese *db/db* mice after four weeks of feeding. The lipid content of dried Susabinori (600 g) was quantified as 26.5 g (4.4%), comprising major fatty acids such as EPA (62.4%) and palmitic acid (24.7%) [77]. Ongoing research utilizing genome-wide impact studies through RNA sequence analysis of the hepatic transcriptome has identified 15 genes that are upregulated following the administration of a diet enriched with *N. yezoensis* lipids in obese *db/db* mice. Subsequent analysis indicated that *a* lipid-rich diet might influence the metabolomics of arachidonic acid and linoleic acid, which in turn improves hepatic steatosis. These genes may serve as potential targets for the treatment of non-alcoholic fatty liver disease [78]. 

A separate study demonstrated that the liposoluble fraction abundant in polyunsaturated fatty acids (PUFA) extracted from the *N. yezoensis* exhibits hepatoprotective effects against alcohol-induced hepatic injury in vivo. The chemical composition of the extract was analyzed, and it was found to contain total fatty acids (84.52%), β-carotene (10.01%), and sterols (2.30%). The predominant fatty acids in the total fatty acid composition are EPA (54.12%), palmitic acid (21.07%), tricosanoic acid (6.43%), and linoleic acid (4.99%). Treatment with lipid-rich extract increases the activity of antioxidant enzymes, minimizes liver tissue damage, and modulates the expression of proteins involved in the anti-apoptotic signaling pathway [27]. These studies indicate the protective effects of fatty acid components derived from the *N. yezoensis.* Further research is required to assess the roles of different signaling molecules. 

Similarly to fatty acids, one research team evaluated the protective effects in vivo of glycoprotein from *N. yezoensis* against chronic ethanol-induced hepatic toxicity [79] and d-galactosamine (d-GalN)/LPS-induced acute liver failure [80]. The available data shows that d-GalN and LPS are frequently employed to generate inflammatory responses and oxidative stress in the liver. In both models, glycoprotein supplementation demonstrated protective effects, primarily through the restoration of the antioxidant system, reduction in inflammatory markers, and downregulation of MAPKs [79,80]. 

Dysbiosis of microbial composition due to a poor diet is regarded as a significant indicator of metabolic diseases. Researchers have assessed the impact of several bioactive compounds on dysbiosis of the gut microbiota. A recent study revealed the advantageous effects of porphyran derived from *N. yezoensis* (PYP) on fruit fly (*Drosophila melanogaster*) third instar larvae stage on a high-sucrose diet *via* altering their gut microbiota makeup. PYP supplementation ameliorated metabolic dysfunction in high sucrose-fed larvae, as indicated by the significantly increased expression of Drosophila insulin-like peptides 2, 3, or 5 and decreased expression of the inflammatory factor Upd3 (IL-6 cytokine of *Drosophila*). Moreover, PYP suppressed the relative abundance of the *Fusobacterium*, *Catenibacterium,* and *Escherichia*-*Shigella* by modulating the abundance of beneficial microbes, specifically *Lactobacillus*, *Akkermansia*, *Bacillus*, *Geobacillus*, *Aneurinibacillus*, and *Corynebacterium*. The study demonstrated the functional prebiotic effects of a porphyran derived from *N. yezoensis* [81] (Table 10).

### 4.11. Anti-Osteoarthritis

Osteoarthritis (OA) is a degenerative disease that increases with age. A recent study reported the anti-osteoarthritic effects of a fermented alcohol extract (30%) of *N. yezoensis* (FEPY), both in vitro and in vivo. The pretreatment with FEPY downregulated expression of inflammatory markers and cartilage-degrading enzymes mediated by MAPK and NF-kB signaling pathways. This study indicates that the FEPY (30%) exerts anti-osteoarthritic effects in chondrocytes induced by IL-1β and DMM-induced osteoarthritis rat model. Future research should focus on evaluating the bioactive components from *N. yezoensis* for their potential anti-osteoarthritic effects [83] (Table 11).

### 4.12. Kidney Stone Treatment

**Figure 5:**
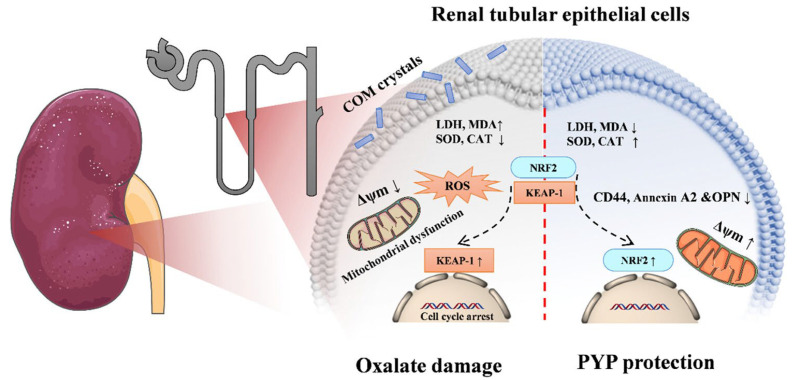
Schematic representation of the mechanism of inhibition of calcium oxalate stones by PYP. Reproduced with kind permission from [84]. Copyright © 2024 American Chemical Society.

High oxalate conditions have been shown to trigger the development of calcium oxalate (CaOx) crystals both in vitro and in vivo. A recent study demonstrated the protective effects of *N. yezoensis* polysaccharides (PYP) against oxalate-induced kidney injury by assessing oxidative damage, adhesion molecule expression, and intracellular organelle integrity. PYP supplementation improved kidney function and inhibited kidney stone formation, elucidating its underlying molecular mechanisms (Figure 5). Specifically, PYP treatment upregulated antioxidant proteins such as SOD, Nrf2, HO-1, and CAT, thereby activating the Nrf2/Keap1 signaling pathway [84] (Table 11). 

### 4.13. Anti-Allergic 

Recent research has focused on anti-allergic food factors owing to their minimal side effects. Allergy, commonly referred to as hypersensitivity, is categorized as type I to type IV based on the mechanisms of tissue injury [89]. Polysaccharide from *N. yezoensis* f. *narawaensis* (PPY) exerted anti-allergic effects against type I hypersensitivity, with increased IL-10 levels in the blood following oral administration after 4 days of intervention [85]. A subsequent investigation suggested that PPY exerts its anti-allergic effects via IL-10 production in intestinal epithelial cells, facilitated by NADPH oxidase-mediated H_2_O_2_ production [86]. Further cell and animal studies are needed to determine the anti-allergic effects of *N. yezoensis* strains from different geographical locations (Table 11). 

### 4.14. Protection Against Vascular Calcification 

**Figure 6:**
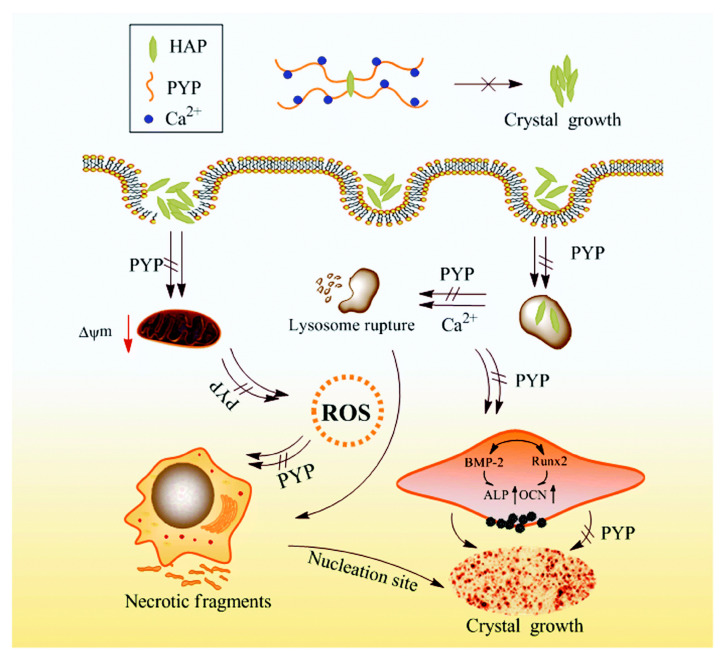
Schematic of the mechanism by which PYPs inhibit cell injury and calcification caused by HAP. Reproduced with kind permission from [87]. Copyright 2020, Royal Society of Chemistry (RSC).

Vascular calcification is a disease characterized by the accumulation of hydroxyapatite (HAP) crystals in vascular smooth muscle cells (VSMCs). This calcification harms cells and induces VSMCs to adopt an osteogenic phenotype, resulting in increased arterial stiffness and elevated risk of cardiovascular disease, particularly in individuals with chronic kidney disease (CKD). Huang et al. [87] evaluated the protective effects of polysaccharides (PYP1, PYP2, PYP3, and PYP4) with different molecular weights (576 kDa, 49.5 kDa, 12.6 kDa, and 4.02 kDa, respectively) against damage in A7R5 (fibroblast-like) cells triggered by HAP. PYP treatment improved cell viability, lowered ROS levels and lactate dehydrogenase release, and reduced cell necrosis. Moreover, PYP supplementation markedly diminished the adhesion and internalization of HAP crystals by cells and suppressed the osteogenic transformation of A7R5 cells (Figure 6). 

Among all, the lower-molecular-weight polysaccharide demonstrated superior activity. Additionally, PYP4 treatment significantly inhibited calcium deposition in mouse aortic tissue of adenine-induced chronic renal failure mice. This study highlights the therapeutic importance of polysaccharides, mainly low molecular weight, for treating and preventing vascular calcification associated with cardiovascular and chronic kidney diseases [87].

### 4.15. Antivirus

Coronavirus disease (COVID-19), triggered by severe acute respiratory syndrome coronavirus 2 (SARS-CoV-2), has imposed a considerable burden on public health and social security. Although the World Health Organization rescinded the emergency status of COVID-19 in May 2023, researchers are pursuing more sustainable remedies for this deadly virus, with bioactive compounds derived from seaweed as promising alternatives. Accordingly, the bioactive component, oligo-porphyran (21 kDa, OP145), was extracted from *N. yezoensis* and its inhibitory activity was assessed against SARS-CoV-2 both in vitro and in silico. As analyzed using Surface plasmon resonance analysis indicated that OP145 binds to the spike glycoprotein (proteins). Moreover, SARS-CoV-2 pseudovirus neutralization test confirmed that OP145 could inhibit the S-protein binding with ACE2 receptor of the target cell (Opti- HEK293/ACE2 cells) with an EC_50_ of 37.52 μg/mL (Table 11). Molecular docking simulations also showed a connection between the main component of OP145 and the S-protein, which was corroborated by in vitro results. Additional in vitro and in vivo studies are required to evaluate the potential of OP145 [88].

### 4.16. Antibacterial 

The incidence of antimicrobial resistance is rapidly increasing, prompting researchers to seek sustainable and effective alternatives. A previous study has documented the antibacterial activity of recombinant cyclophilin prepared by expressing cyclophilin genes from *N. yezoensis* (PyCyp) in *E. coli*. The recombinant protein was characterized by UV-Vis spectrophotometry and circular dichroism (CD) spectroscopy, which demonstrated its ability to catalyze cis-trans isomerization. The recombinant protein exhibited inhibitory activity against both Gram-positive and Gram-negative bacteria (Table 11). The highest antibacterial activity (86% inhibition) was reported against *Pseudomonas aeruginosa,* which was comparable to that of the standard antibiotic drug, chloramphenicol. A dye-binding assay also demonstrated the permeabilization ability of PyCyP in *Staphylococcus aureus,* further confirming the damage caused by pathogenic bacteria. PyCyp also exhibited antioxidant activity. This study showed that recombinant proteins have the potential to be utilized as therapeutics, and future research should be conducted to evaluate for other biological activities [22].

## 5. Conclusions 

*N. yezoensis* is extensively cultivated in East Asia, specifically Korea, China, and Japan, owing to its importance as a raw material in the food and processing industry. *N. yezoensis* is widely used because of its ecological and therapeutic importance. Multiple studies have corroborated the existence of many functional components, specifically polysaccharides, proteins, peptides (synthetic and recombinant), MAAs, porphyran, and fatty acids. The health-promoting properties of *N. yezoensis* have been supported by in vitro and in vivo studies, underscoring its antioxidant, anti-inflammatory, neuroprotective, anti-aging, anti-colitis, antibacterial, anti-osteoarthritic, anti-atopic dermatitis, anti-metabolic disorder, protection against kidney stone development and vascular calcification, anti-atrophy, and antivirus effects. While a few clinical studies have suggested potential benefits in humans, larger controlled human trials are needed to confirm the efficacy, determine optimal doses, and ensure safety. Low-molecular-weight derivatives of polysaccharides and high-molecular-weight derivatives of porphyrans have been shown to have superior biological activity. Furthermore, typical *N. yezoensis*, together with discolored nori, has proven to be an excellent source of antioxidant and anti-inflammatory constituents. Future research should prioritize animal and clinical studies to gain a comprehensive understanding of the bioactive ingredients in *N. yezoensis*. Studies comparing nutritional profiles based on geographical and varietal differences are limited and require further exploration. *N. yezoensis* is distinguished by its rich nutritional profile and diverse bioactivities, which make it a valuable component in the development of functional foods and nutraceuticals. 

## Figures and Tables

**Table 1:** Fatty acid composition of *N. yezoensis*.

Compound Name	Composition (%)	Jianghaida No. 1 (%) *	Sutong No. 1 (%) *
Octanoic acid (C8:0)	0.01	--	--
Lauric acid (C12:0)	--	0.03	0.06
Myristic acid (C14:0)	1.26	0.20	0.25
Pentadecanoic acid (C15:0)	1.44	0.22	0.24
Palmitic acid (C16:0)	21.07	41.74	43.01
Palmitoleic acid (C16:1)	1.63	0.32	0.32
Heptadecanoic acid (C17:0)	--	0.08	0.10
Stearic acid (C18:0)	0.48	1.11	1.14
Oleic acid (C18:1(n-9), cis)	1.10	2.44	2.46
Alpha-linoleic acid (C18:2(n-6), cis)	4.99	1.88	1.86
Linolenic acid (C18:3(n-3))	0.34	0.28	0.3
Gamma-linolenic acid (C18:3(n-6))	0.61	0.22	0.2
Arachidic acid (C20:0)	--	0.06	0.06
Cis-11-eicosenoic acid (C20:1)	0.33	5.77	5.73
Cis-11,14-eicosadienoic acid (C20:2)	0.40	1.35	1.30
Cis-11,14,17-eicosatrienoic acid (C20:3 (n-3))	0.03	0.47	0.15
Cis-8,11,14-eicosatrienoic acid (C20:3 (n-6))	2.75	2.14	1.99
Cis-5,8,11,14-eicosatetraenoic acid (C20:4 (n-6))	0.67	1.85	1.75
Cis-5,8,11,14,17-eicosapentaenoic acid (C20:5 (n-3))	54.12	36.52	36.00
Heneicosanoic acid (C21:0)	--	0.02	0.03
Behenic acid (C22:0)	--	0.05	0.04
Erucic acid (C22:1(n-9))	0.32	1.01	0.93
Cis-13,16-docosadienoic acid (C22:2)	1.70	--	--
Docosahexaenoic acid (C22:6n-3)	--	0.08	0.13
Tricosanoic acid (C23:0)	6.43	1.85	1.75
Lignoceric acid (C24:0)	0.35	--	--
Nervonic Acid (C24:1)	--	0.33	0.21

* Fatty acid composition of two varieties of head water *N. yezoensis*.

**Table 2:** Antioxidant activity of *N. yezoensis* in cell and animal models.

Test Material	Experimental Model	Outcomes/Mechanisms	Ref.
Polyphenols and protein-rich extracts (PP)	Vero cells + PP-rich extract (12.5, 25, and 50 μg/mL) 1 h + post AAPH (10 mM) treatment, 24 h	↓ ROS levels, Dose-dependent reduction in apoptotic bodies	[25]
	Zebrafish embryos PP-rich extract Sinan (12.5, 25, and 50 μg/mL) 1 h + post AAPH (15 mM, 25 μL) treatment, 24 h	↓ ROS levels, ↓ lipid peroxidation	
13 synthetic peptides (PBP 1–13)	HepG2 cells + PBP 1–13 (1 µg/mL), + H_2_O_2_ (5 mM), 1 h	↓ ROS levels, ↑ p-Nrf2, ↑ SOD, ↑ cells’ survival, ↓ apoptosis	[31]
SGID protein hydrolysate	ORAC	SGID = 432.763 mM TE/mg, Unhydrolyzed protein (UP) = 106.03 mM TE/mg	[32]
	ABTS	SGID= 64.42 mM TE/mg UP =12.44 mM TE/mg	
	DPPH	SGID = 58.99% UP = 10.86%	
	Superoxide radical	SGID = 47.65%, UP= 7.8%	
	Hydroxyl radical	SCID=61.63%, UP = 8.3%	
	NO assay: HepG2 + (5, 25, 50, and 100 µg/mL), APAP (15 mM) 37 °C, 18 h	↓ NO concentration-dependent	
*N. yezoensis* fermented using *B. amyloliquefaciens* MMB-02, *L. plantarum* L13, *S. cerevisiae* A8	DPPH	F = 54.87%, control (C) = 14.68%	[33]
	ABTS	F = 57.39%, C = 21.82%	
	FRAP value	F (Fe^3+^ equivalents) = 1.43, C = 0.61	
	SOD, CAT, and GSH levels	SOD (U/mL): F= 17.08, C = 4.28, CAT (U/mL): F = 0.072, C = 0.046, GSH (μmol/L) F = 28.15, C = 14.69	
Resistant strain (G1), commercial strain (CP)	293T cells + CP or G1 (250, 500, and 1000 µg/mL), 3 h + H_2_O_2_ (600 µM, 600 µL), 1 h DCFH-DA, apoptosis (H_2_O_2_, 600 µM)	↓ ROS levels, Pro-apoptotic genes: ↓ P53, ↓ Bax, ↓ caspase-3, ↑ Bcl-2, G1 more effective than CP	[34]
Recombinant PPI protein	HepG2 cells + PPI protein (0.001, 0.01, 0.1 and 1 µg/mL) + H_2_O_2_ (1 mM, 1 h)	↓ ROS, ↑ CAT, ↑ GPx, ↑ SOD, ↑ TRR activities and expressions	[35]
*N. yezoensis* polysaccharides (PYPS)	DPPH (2, 4, 6, 8, and 10 mg/mL)	Dose-dependent effect 28.60 % to 49.40 %	[36]
	ABTS (2, 4, 6, 8, and 10 mg/mL)	76.19% (10 mg/mL)	
	Hydroxyl (2, 4, 6, 8, and 10 mg/mL)	50.05 % (2 mg/mL) and 59.52 % (4 mg/mL)	

AAPH: 2,2′-azobis (2-amidinopropane) dihydrochloride; ABTS: (2,2′-azino-bis (3-ethylbenzothiazoline-6-sulfonic acid)); APAP: acetaminophen; Bax: Bcl2 associated X protein; Bcl-2: B-cell lymphoma 2 protein; CAT: catalase; DPPH: 2,2-diphenyl-1-picrylhydrazyl; GPx: glutathione peroxidase; H_2_O_2_: hydrogen peroxide; NO: nitric oxide; ORAC: Oxygen radical absorbance capacity; p-Nrf2: phosphorylated nuclear factor erythroid-derived 2-like 2; PBP: phycobiliprotein; PPI: cyclophilin-type peptidylprolyl cis-trans isomerase; ROS: reactive oxygen species; SGID: simulated gastrointestinal-digested; SOD: superoxide dismutase; TRR: thioredoxin reductase. *B. amyloliquefaciens*: *Bacillus amyloliquefaciens*, *L. plantarum*: *Lactiplantibacillus plantarum*, *S. cerevisiae*: *Saccharomyces cerevisiae*. ↑ = increases, and ↓ = decreases.

**Table 3:** Anti-inflammatory activity of porphyran extracted from *N. yezoensis*.

Test Material	Experimental Model	Outcomes/Mechanisms	Ref.
Porphyran	RAW264.7 cells + porphyran (250 and 500 µg/mL), 1 h + LPS (2 ng/mL)	No cytotoxicity, ↓ NO, ↓ iNOS, ↓ NF-κB	[38]
Porphyran (D1, D2, D3, D4)	RAW264.7 cells + porphyrans (250, 500, and 1000 µg/mL), 1 h + LPS (20 ng/mL) 24 h	No cytotoxicity, D2 porphyran = ↓ NO, ↓ iNOS, ↓ TNF-α ↓ Osteoclastogenesis	[39]
Porphyran	PBMCs + porphyran pre-treatment (10, 25, 50, and 100 µg/mL), 1 h + LPS (20 ng/mL), 24 h	↓ IL-1ꞵ, ↓ IL-6, ↓ IL-12p70, ↓ TNF-α, ↓ IFN-ϒ	[40]
	Differentiated mature MODCs, porphyran pre-treatment (10, 25, 50, and 100 µg/mL), 1 h + LPS (20 ng/mL), 24 h	↓ IL-12p70, ↓ TNF-α, ↓ IL-6, ↓ CD40, ↓ CD80, ↓ Cd86, ↓ CCR7	
	PBDCs + porphyran pre-treatment (50 µg/mL), 1 h + LPS (20 ng/mL), 24 h	↓ CD40, ↓ CD80, ↓ Cd86, ↓ MHCI, ↓ MHCII, suppressed proliferation/activation of CD4 T cells	
Porphyran	Bone marrow-derived dendritic (BMDC) from C57BL/6 mice, porphyran pre-treatment (0, 10, 25, 50, and 100 μg/mL), 1 h + LPS (20 ng/mL)	Inhibited BMDCs activation ↓ CD40, ↓ CD80, ↓ Cd86, ↓ CCR7, ↓ IL-6, ↓ TNF-α, ↓ IL-12p70 (dose-dependent)	[41]
	C57BL/6 mice + porphyran pre-treatment (i.p., 12.5, 25, 50, and 100 mg/kg), 1 h + LPS (i.p., 100 μg/kg)	↓ spleen dendritic cells ↓ CD40, ↓ CD80, ↓ Cd86, ↓ CCR7 ↓ IL-6, ↓ TNF-α, ↓ IL-12p70 (dose-dependent) ↓ CD4T, ↓ CD8T, ↓ IFN-ϒ, ↓ T-bet	

CD: cluster of differentiation; IFN-ϒ: interferon gamma; IL: interleukin; iNOS: inducible nitric oxide synthase; LPS: lipopolysaccharide; MHC: major histocompatibility complex; IP: intraperitoneal; MODCs: monocyte-derived dendritic cells; NO: nitric oxide; PBDCs: peripheral blood dendritic cells; CCR7: C-C chemokine receptor type 7; PBMCs: peripheral blood mononuclear cells; TNF-α: tumor necrosis factor-alpha. ↑ = increases and ↓ = decreases.

**Table 4:** Studies showing neuroprotective activity of bioactive components extracted from *N. yezoensis*.

Test Material	Experimental Model	Outcomes/Mechanism	Ref.
Phycoerythrin-derived tryptic peptide (PYP)	Primary rat hippocampal neurons+ glutamate (50–200 μM) or PYP (0.25–2 μg/mL), 24 h	↓ GRP78, ↓ ER stress, ↓ SA-β-gal, ↓ neurite dysregulation, ↓ JNK, Activation of TrkB-PI3K-ERK1/2 signaling	[43]
PYP	Rat prefrontal cortex + PFOS (25–400 µM) + PYP (0.25–2 µg/mL), 24 h	↓ GRP78, ↓ JNK,↓ ER stress, Activated TrkB-PI3K-ERK1/2 signaling, ↓ calcium levels	[44]
Oligo-porphyran (OP)	PC12 cells + OP (50, 100, 200 μg/mL), 24 h + 6-OHDA (100 μM), 24 h + 30 min DCFH-DA assay	↓ apoptosis, ↑ MMP, ↓ ROS levels, ↑ SOD, ↑ GSH, ↓ Bax/Bcl-2, ↓ cytochrome c, ↑ TH, ↑ DAT, ↓ TNF-α, ↓ IL-1β, ↓ IL-6	[45]

6-OHDA: 6-hydroxydopamine hydrochloride; Bcl-2: B-cell lymphoma 2 protein; Bax: Bcl-2-associated X protein; DAT: dopamine transporter protein; ER: endoplasmic reticulum; ERK: extracellular signal-regulated kinase; GRP78: glucose-regulated protein 78; GSH: glutathione; IL: interleukin; JNK: c-Jun N-terminal kinase; MMP: Mitochondrial membrane potential; PFOS: perfluorooctane sulfonate; PI3K: phosphatidylinositiol 3-kinase; ROS: reactive oxygen species; SA-β-gal: senescence-associated β-galactosidase; SOD: superoxide dismutase; TH: tyrosine hydroxylase; TNF-α: tumor necrosis factor-alpha; TrkB: tropomyosin-related kinase B. ↑ = increases and ↓ = decreases.

**Table 5:** Anticancer activity of bioactive constituents extracted from *N. yezoensis*.

Test Material	Experimental Model	Outcomes/Mechanisms	Ref.
Porphyran (PYP) and its derivatives	Hep3B, HeLa, and MDA-MB-231 cells + PYP, PYP-20, PYP-50 (200 μg/mL), 48 h	↓ HeLa viability- PYP: 75%, PYP-50: 50%, PYP-20: 50%. ↓ Cyclin B1, ↓ CDK1, ↑ p53, ↑ p21. Blocking of G2/M phase of HeLa cell cycle	[47]
		↓ MDA-MB-231 viability- PYP-50: 42%, PYP-20: 42%	
		Hep3B viability- PYP: 80%, PYP-50: 25%, PYP-20: 40%	
*N. yezoensis* Sookwawon 104 polysaccharides (PYSP) and derivatives	HeLa, Hep3B and MDA-MB-231 cells + PYSP, PYSP-20 or PYSP-100 (200, 500 µg/mL), 48 h	Antiproliferative activity. ↓ Cyclin B1, ↓ Cdk1, ↑ P53, ↑ P21	[48]
Sulfate polysaccharide, Galactan (GPY)	DU145 and PC-3 cells + GPY_crude_, GPY_300_, or GPY_10_, 50 µL of 10 µmol/L DCFH-DA, 24 h	↓ DU145 cell viability: GPY_crude_: 64%, GPY_10_: 80%, GPY_300_: 68%. ↑ ROS in DU145-GPY_10_: ↓ SOD2, ↑ apoptosis in DU145- GPY_10_: ↑ Bax, ↑ caspase 9, ↑ caspase 8, ↑ caspase 3, ↓ PI3K, ↓ Akt, ↓ mTOR	[49]
		↓ PC-3 cell viability: GPY_10_: 73%. DU145 was more sensitive to GPY_10_	

CDK1: cyclin-dependent kinase 1; mTOR: mammalian target of rapamycin; p21: a cyclin-dependent kinase inhibitor; p53: tumor suppressor protein; PI3K: phosphatidylinositiol 3-kinase; ROS: reactive oxygen species; SOD2: superoxide dismutase 2. ↑ = increases and ↓ = decreases.

**Table 7:** Anti-aging effects of extract and bioactive constituents of *N. yezoensis*.

Test Material	Experimental Model	Outcomes/Mechanisms	Ref.
Mycosporine-like amino acid (Porphyra-334)	Human skin fibroblasts (CCD-986sk) + UVA light irradiation (10 J/cm^2^) + porphyra-334 (10, 20, and 40 μM), 24 h	↓ intracellular ROS, ↓ SA-β-gal, ↓ MMP-1, ↑ procollagen secretion, ↑ COL1A1, ↑ elastin	[54]
Peptide (PYP1–5)	Human dermal fibroblast cells (Hs27) + PYP1–5 (250, 500 and 1000 ng/mL), 24 h	↓ MMP-1, ↑ TIMP-1, ↑ TIMP-2, ↑ elastin, ↑ COL1A1, ↑ COL1A2, ↑ TGF-β1, ↑ p-Smad3, ↑ p-Smad2, ↓ Smad7 (inhibitor), ↑ Sp1	[55]
*N. yezoensis* extract	Mouse melanocytes (Melan-A), human dermal keratinocytes (HaCaT cells), human dermal fibroblasts (1064 SK) + extract (100, 200, 400, and 800 μg/mL)	↓ melanin content: 400 μg/mL (44.1%), 800 μg/mL (53.8%), ↓ tyrosinase activity at 800 μg/mL (35.5%). ↓ melanogenic enzymes: MITF, TRP-1, TRP-2, ↑ type I procollagen, ↓ MMP-2, ↓ MMP-9.	[56]
**Clinical Application**			
Lotion with *N. yezoensis* extract (0.1% w/w)	23 subjects, 4 weeks and 8 weeks	↑ Skin brightness, 8 weeks, extract = 1.32% control 0.46% (*p* < 0.05). ↓ melanin content = 2.4% (4 weeks) and 3.0% (8 weeks), control no change (*p* < 0.05). ↑ skin-lightening effect = (*p* < 0.05).	

COL1A1/A2: collagen type I alpha A1 chain/A2 chain; MITF: microphthalmia-associated transcription factor; MMP-1: matrix metalloproteinase-1; ROS: reactive oxygen species; SA-β-gal: senescence-associated β-galactosidase; Sp1: specificity protein 1; TGF-β: transforming growth factor-beta; TIMP: tissue inhibitor of metalloproteinase; TRP-1/2: tyrosinase-related protein-1/2. ↑ = increases and ↓ = decreases.

**Table 8:** Induction of cell proliferation by *N. yezoensis* peptides.

Test Material	Experimental Model	Outcomes/Mechanisms	Ref.
*N. yezoensis* peptide (PY-PE)	IEC-6 cells + PY-PE (1000, 500, 250, and 125 pg/mL), 24 h	↑ IGF-IR, ↑ IRS-1, ↑ Shc, ↑ py99, ↓ JNK, ↓ p38, ↑ ERK1/2, ↑ p85, ↑ p110, ↑ PDK1, ↑ c-Jun, ↑ p-Akt, ↑ c-fos c	[61]
*N. yezoensis* peptide [PYP1 (1–20)]	IEC-6 cells + PYP1 (1–20) (1000, 500, 250, and 125 pg/mL)	↑ p-EGFR, ↑ Shc, ↑ Grb2, ↑ Sos, ↑ Ras, ↑ Raf, ↑ MEK, ↑ p-ERK, ↑ cell cycle progression, ↑ cyclin D1 and E, ↓ p21, ↑ Cdk4, ↑ Cdk2, ↑ Cdk6, ↑ pRb, ↓ p27	[62]
Recombinant cyclophilin (pyCyp)	IEC-6 cells + pyCyp (50, 25, and 5 pg/mL), 48 h	↑ p-EGFR, ↑ Sos1,↑ Grb2,↑ Ras, ↑ p-Raf1, ↑ p-MEK, ↑ p-ERK, ↑ cyclin A, ↑ cyclin E, ↑ Cdk2, ↑ Cdc25a, ↑ pRb, ↑ p-pRb, ↓ p27, ↓ p21	[63]

Cdc25a: cell division cycle 25a; CDK1: cyclin-dependent kinase 1; Cdk2: cyclin dependent kinase 2; Grb2: growth factor receptor-bound protein-2; IEC: Intestinal epithelial cell; p21: a cyclin-dependent kinase inhibitor; p53: tumor suppressor protein; p-EGFR: phosphorylated-epidermal growth factor receptor; p-ERK: phosphorylated extracellular signal–regulated kinases; p-MEK: phosphorylated mitogen-activated protein kinase/ERK kinase; p-pRb: phosphorylated retinoblastoma; p-Raf-1: phosphorylated rapidly accelerated fibrosarcoma-1; Ras: renin-angiotensin system; Sos: son of sevenless. ↑ = increases and ↓ = decreases.

**Table 10:** Metabolic health-promoting effects of bioactive constituents extracted from *N. yezoensis*.

Test Material	Experimental Model	Outcomes/Mechanisms	Ref.
Porphyran (PPYP)	HepG2 cells+ palmitic acid (25, 50, 100, 200 μM) + PPYP (200 μM), 48 h	↓ TG, ↓ SREBP, ↓ ACC, ↓ FAS, ↑ CPT1, ↑ PPARα	[71]
	*D. melanogaster* larvae + high sucrose (1 M sucrose) + PPYP (25 mg/mL)	↓ TG, ↓ SREBP, ↓ FAS, ↑ Acox57D-d, ↑ FABP	
Porphyran	Male ICR mice+ regular diet group, HFD group, treatment groups: HFD, + porphyran (50, 100, and 200 mg/kg daily), Zhibituo group: HFD + Zhibituo (42 mg/kg daily), 4 weeks	↓ BW gain, Serum: ↑ HFD, ↓ TG, ↓ TC, ↓ LDL-C Fecal: ↑ TC, ↑ TG, effective dose 200 mg/kg. Liver: ↓ liver weight reduction, ↓ TC, ↓ TG, ↓ ALP, ↓ AST, ↓ ALT	[72]
*N. yezoensis* protein (PYP)	SD rats + PYP (100 mg/kg), 2 weeks + AAP (700 mg/kg BW) intraperitoneal injection, 24 h	↓ GOT, ↓ GPT, ↑ GSH, ↓ caspase-3 activity and DNA fragmentation in liver tissue	[73]
Synthetic peptide PYP1 (1–20)	Chang liver cell line (CCL-13) + PYP1 (1–20) (250 or 500 ng/mL) + acetaminophen (15 mM), 24 h	No cytotoxicity, Recovered viability of acetaminophen-triggered cells	[74]
Recombinant peptides (PYP1-AC, PYP1, PYP1-B), and synthetic peptide (SP)	Chang liver cell line (HPV-18) + PYP1-AC, PYP1, PYP1-B, and SP (125, 250, 500, and 1000 pg/mL) + acetaminophen (15 mM), 24 h	No cytotoxicity, ↑ cell viability	[75]
Peptide PYP1–4	HepG2 cells + acetaminophen (15 mM) + PYP1–4 (125, 250 and 500 ng/mL)	↓ NO, ↓ ROS, ↑ HO1, ↑ CAT, ↑ SOD2, ↑ NQO1, ↓ p-JNK ↓ p-p38. ↑ nuclear translocation of Nrf2, ↑ p-GSK3β, ↑ p-Akt, ↑ p-AMPK. ↓ apoptosis, ↓ Bad, ↑ Bcl-2, ↑ Bid, ↑ caspase-3, ↑ caspase-9, ↑ IGF-IR, ↑ EGFR, ↑ IRS-1, ↑ PI3Kp85, ↑ PTEN, ↑ p70S6K, ↑ eIF4E, ↑ GRB2, ↑ p-Akt/Akt, ↑ SHC, ↑ SOS, ↑ p-mTOR/mTOR, ↑ p-MEK/MEK, ↑ p-ERK/ERK	[76]
Susabinori lipid	C57BL/6J mice (normal group), db/db mice, Control diet group, Susabinori lipids diet, 2%, 4 weeks	↓ Liver TG, ↑ adiponectin, ↓ FAS, ↓ malic enzyme, ↓ MCP1, ↑ PPARδ, ↓ ACC1, ↓ SCD1, ↓ SREBP1c	[77]
Lipid extraction from Susabinori powder using chloroform: methanol (V/V = 2:1)	C57BL/6J mice (normal group), male BKS.Cg- +Leprdb/+Leprdb/Jcl (*db*/*db*) mice control diet group and Susabinori lipids diet, 2%, 4 weeks	Hepatic fatty acid content: ↓ LA, ↓ DGLA, ↓ GLA, ↓ AA, ↑ EPA. 15 genes upregulated. AA and LA metabolism-related genes. ↓ Magl, ↓Fabp4	[78]
Polyunsaturated fatty acids -rich extract (PYLP) (using 70% ethanol)	Male BALB/c mice, normal group, alcohol group (alcohol 3 g/kg + saline, 100 μL each), PYLP + alcohol group (PYLP 25 mg/kg + alcohol 3 g/kg mice), silymarin + alcohol group (silymarin 50 mg/kg + alcohol 3 g/kg)	↓ hepatic damage and degeneration. In serum: ↓ GOT, ↓ GPT, ↓ total cholesterol, ↓ MDH In liver: ↑ SOD, ↑ GPx, ↑ CAT, ↓ TBARS Anti-apoptosis: ↓ p53, ↓ Bax, ↑ Bcl-xL	[27]
*N. yezoensis* glycoprotein (PYGP)	SD rats, CON group; ethanol group, ethanol (20%) 3.7 g/kg/BW; ethanol+ PYGP groups, ethanol (20%) + PYGP (150 and 300 mg/kg/BW), 30 days	↓ GOT, ↓ GPT, ↑ GSH, ↑ GSH-px, ↑ CAT, ↓ CYP2E1, ↓ iNOS, ↓ p-p38, ↓ COX-2, ↓ p-JNK, ↓ p-ERK	[79]
PYGP	SD rats, control group; D-GalN (500 mg/kg/BW) + LPS (10 µg/kg/BW) group. D-GalN + LPS + PYGP (150 and 300 mg/kg/BW) groups. PYGP once a day, 7 days	↓ GOT, ↓ GPT, ↓ TBARS, ↑ GSH, ↑ GST, ↑ CAT, ↓ p-JNK, ↓ p-ERK, ↓ p-p38, ↓ COX-2, ↓ iNOS	[80]
Porphyran (PYP)	*D. melanogaster w^1118^* embryos + Control group, HS group: Sucrose (350 g/kg), PYP group: Sucrose (350 g/kg) + PYP (15, 25, and 50 g/kg)	↓ TG, ↓ circulating sugars (25 or 50 g/kg), ↑ Dilp2, ↑ Dilp3, ↑ Dilp5, ↓ Upd3. Modulated gut microbiota	[81]

AA: arachidonic acid; AAP: acetaminophen; ACC1: acetyl-CoA carboxylase 1; Acox57D-d: acyl-Coenzyme A oxidase at 57D distal; ALP: alkaline phosphatase; ALT: alanine transaminase; AMPK: protein kinase AMP-activated catalytic subunit α2; AST: aspartate transaminase; Bad: The Bcl-2-associated death promoter; Bcl-2: B-cell leukemia/lymphoma 2; Bid: BH3 interacting domain death agonist; BW: body weight; CAT: catalase; COX-2: cyclooxygenase-2; CPT1: carnitine palmitoyl transferase 1; CYP2E1: cytochrome P450 2E1; DGLA: dihomo-gamma linoleic acid; Dilp: Drosophila insulin-like peptide; EGFR: epidermal growth factor receptor; elF4E: eukaryotic translation initiation factor 4E; EPA: eicosapentaenoic acid; ERK: extracellular signal-regulated kinase; Fabp4: fatty acid-binding protein 4; FAS: fatty acid synthase; GLA: gamma linoleic acid; GOT: glutamic-oxaloacetic transaminase; GPT: glutamic pyruvic transaminase; GRB2: growth factor receptor bound protein 2; GSH: glutathione; GSH-px/GPx: glutathione peroxidase; GSK3β: glycogen synthase kinase 3β; GST: glutathione S-transferase; HFD: high-fat diet; HO1: heme oxygenase 1; ICR: Institute of Cancer Research; IGF-IR: insulin-like growth factor 1 receptor; iNOS: inducible nitric oxide synthase; IRS-1: insulin receptor substrate 1; JNK: c-jun N-terminal kinase; LA: linoleic acid;; Magl: monoacylglycerol lipase; MCP1: monocyte chemoattractant protein-1; MDH: malondialdehyde; MEK: mitogen-activated protein kinase kinase; NO: nitric oxide; NQO1: quinone oxidoreductase 1; Nrf2: nuclear factor erythroid 2 like 2; p: phosphorylated; p38: p38 MAP kinase; p70S6K: p70S6 kinase; PPARα: peroxisome proliferator activated receptor alpha; PPARδ: peroxisome proliferator-activated receptor gamma; PTEN: phosphatase and tensin homolog; ROS: reactive oxygen species; SCD1: stearoyl-CoA desaturase 1; SD: Sprague–Dawley; SHC: SHC adaptor protein 1; SOD2: superoxide dismutase 2; SOS: SOS Ras/Rac guanine nucleotide exchange factor 1; SREBP1c: sterol regulatory element-binding protein 1c; TBARS: thiobarbituric acid reactive substances; TC: total cholesterol; TG: triglyceride.↑ = increases and ↓ = decreases.

**Table 11:** Different therapeutic properties of extracts and bioactive constituents of *N. yezoensis*.

Test Material	Experimental Model	Outcomes/Mechanisms	Ref.
Anti-osteoarthritic			
Fermented ethanolic extract of *N. yezoensis* (FEPY)	Primary chondrocytes + pretreatment (30% FEPY: 0.25, 0.5, 1, and 2), 1 h + IL-1β (10 ng/mL), 2 or 24 h.	↓ Nitrite, ↓ PGE2, ↓ iNOS and ↓ COX-2, ↓ MMP13, ↓ MMP3, ↓ MMP1, ↓ ADAMTS5 and ↓ ADAMTS4	[83]
	Sprague-Dawley rats ex vivo: the knees of postnatal rats (5-day-old), post 3 days + pretreatment FEPY (30%), 1 h + IL-1β (10 ng/mL), 48 h	Articular cartilage stain was restored to a darker color. ↑ collagen type II,↑ aggrecan, ↓ p-ERK, ↓ p-JNK, and ↓ p-P38, ↑ NF-κB p65,↓ p-IκB-α	
	Group 1 (normal), group 2 (sham), group 3 (DMM, + normal saline), and groups 4 to 6 (DMM + FEPY 30% (50, 100, and 200 mg/kg BW), 8 weeks	Proteoglycans content OARSI score = DMM surgery group (12); FEPY 50 (7); FEPY 100 (6); FEPY 200 (5), ↑ collagen type II	
Kidney stone treatment			
Polysaccharide from *N. yezoensis* (PYP)	Viability assay: HK-2, NRK-52E, NK-49F, MDCK + PYP (10, 20, 30, 40, 60, 80, 120, 160, 240 μg/mL), 24 h HK-2 cells + oxalate solution (1.0 mM) + PYP (10, 30, and 60 μg/mL), 24 h	PYP > 120 μg/mL was safe ↑ HK-2 cells viability, ↓ ROS, ↓ MDH, ↑ SOD, ↑ CAT, ↑ Nrf2, ↓ keap1, ↓ lactate dehydrogenase release, restored mitochondrial membrane depolarization, protected membrane integrity, ↓ CD44, ↓ OPN, ↓ Annexin A2	[84]
	In vivo distribution assay: C57BL/6 mice Normal group: saline 200 μL daily, 1 week (I.P.) PYP-ICG (I.P.) 200 mg/kg/BW Glyoxylic acid stone modeling group: GA 70 mg/kg/day daily, 1 week (I.P.), PYP-ICG (I.P.) 200 mg/kg/BW. Fluorescence expression was monitored at 2, 4, 8, 24, and 48 h using a small animal imaging system.	fluorescent signal = abdomen mainly disappeared after 48 h	
	Therapeutic evaluation: SD rats Normal group: standard diet Stone group: standard diet + ethylene glycol (1%), 28 days PYP group: ethylene glycol + 50, 200, and 400 mg/kg wt, 28 days,	Prevented CaOx crystal formation, deposition and adhesion risk: ↓ CD44 and ↓ OPN Inhibited renal injury ↓ Kim-1 (renal injury factor), ↑ SOD, ↑ CAT, ↑ Nrf2, ↓ keap1 ↓ IL-6, ↓ MCP-1	
Anti-allergic			
Polysaccharide from *N. yezoensis* f. *narawaensis* (PPY)	Female BALB/c mice, PPY (1 mg/mL), oral dose, 4 days + anti-TNP IgE antibody (IV injection 30 min. 2,4,6-trinitrochlorobenzene 1.6 % (*w*/*v*) in acetone/olive oil (1:1) + anti-IL-10 (100 µg/mL) antibody or irrelevant IgG (100 µg/mL), every 2 days	PCA reaction: inhibited ear edema = 39% Dose-dependent inhibition by PPY ↑ IL-10	[85]
	RBL-2H3 cells or Co-culture system (Caco-2 and RBL-2H3 cells): + PPY (1 or 10 mg/mL) 2 h + anti-DNP IgE (1 μg/mL),1 h + DNP-BSA (100 ng/mL), 1 h	No release of β-hexosaminidase	
Polysaccharide from *N. yezoensis* f. narawaensis (PPY)	PCA reaction: BALB/c mice + PPY (500, 750, 1000 μg, oral dose), 4 days + anti-TNP IgE antibody (2 μg/100 μL, IV), 30 min + 1.6 % (*w*/*v*) picryl chloride in acetone/olive oil (1:1) after 2 h. Active cutaneous anaphylaxis (ACA): PPY (oral dose, 7 days) + adjuvant 300 μL OVA (10 μg) and Al (OH)_3_ (1 mg), I.P., 4 times, 5 days + OVA (5 μg) I.V.	Inhibited ear edema dose-dependent ↑ IL-10 ↓ rectal temperature	[86]
	RBL-2H3 + IL-10 (10 ng/mL) or H_2_O_2_ (0.01, 0.1, 1, 10 μM), 2 h + anti-TNP IgE (200 ng/mL), 2 h + TNP-BSA, 30 min HT-29/RBL-2H3 co-culture system + PPY (10 mg/mL), 2 h on apical side of HT-29 + IL-10 (10 mg/mL)	↓ β-hexosaminidase release together by IL-10 and H_2_O_2_ treatment PPY increased H_2_O_2_ production from HT-29 cells, pretreatment with an NADPH oxidase inhibitor suppressed H_2_O_2_ production, inhibited mast cell degranulation	
Protection against vascular calcification			
Polysaccharides from *N. yezoensis* (PYP1-PYP4)	A7R5 cells + HAP (200 μg/mL) + PYP1-PYP4 (50, 100, 200, and 400 μg/mL), 24 h	Inhibited HAP damage, ↑ cell viability, Protecting effects 200 > 400 > 100 > 50 μg/mL. ↓ LDH release, ↓ ROS. Inhibited decline in mitochondrial membrane potential, Inhibited: intracellular calcium level, Ca deposition, ALP, HAP adhesion and endocytosis. ↓ cell necrosis, Inhibited osteogenic transformation: ↓ BMP-2, ↓ Runx2, ↓ OCN	[87]
	C57BL/6 mice normal group; calcification model group; PYP4 treated groups: 50, 100, and 200 mg kg/day) + adenine 0.2% (*w*/*w*).	↓ aortic calcium level, ↓ creatinine, ↓ phosphate, ↓ BUN	
Antivirus			
Sulfated oligo-porphyran OP145	Pseudovirus packaged by lentivirus vector with the S-protein (25 μL) + OP145 (0.391, 1.563, 6.25, 25, 100 and 400 μg/mL), + ACE2 overexpressed target cells, Opti-HEK293/ACE2, 24 h	Competitively inhibited attachment of S-protein to ACE2	[88]
Antibacterial			
Recombinant cyclophilin (PyCyP)	*E. coli* (KCTC 2571)*, Staphylococcus aureus* (KCTC 1621)*, Pseudomonas aeruginosa* (KCTC 1750)*,* and *Bacillus subtilis* (KCTC 1028) *+* PyCyP (100, 50, 25, 12.5, 6.25, and 3.125, μg/mL)	MIC (μg/mL): PyCyP = *E. coli* (6.25), *S. aureus* (6.25), *B. subtilis* (12.5), and *P. aeruginosa* (3.12), positive control Chloramphenicol (c) = (3.12)	[22]

ACE2: angiotensin-converting enzyme 2; ADAMTS: a disintegrin and metalloproteinase with thrombospondin motif; BW: body weight; COX-2: cyclooxygenase-2; DMM: destabilization of the medial meniscus; ERK: extracellular signal-regulated kinase; HT-29 cells: human intestinal epithelial cell line; IL-1β: interleukin-1beta; iNOS: inducible nitric oxide synthase; IP: intraperitoneal; IV: intravenous; IκB: NF-kappa-B inhibitor alpha; JNK: c-Jun N-terminal kinase; KCTC: The Korean Collection for Type Cultures; MAPK: mitogen-activated protein kinase; MIC: minimum inhibitory concentration; MMP: matrix metalloproteinase; NF-κB: nuclear factor kappa-light-chain-enhancer of activated B cell; NO: nitrite oxide; OARSI: Osteoarthritis Research Society International; p: phosphorylated; PCA: passive cutaneous anaphylaxis; PGE2: prostaglandin E2; RBL-2H3: rat basophilic leukemia cell line; TG: triglyceride; LDH: lactate dehydrogenase; ALP: alkaline phosphatase; GA: glyoxylate; OPN: osteopontin; CD: cluster of differentiation; ROS: reactive oxygen species; CaOx: calcium oxalate; MDH: malondialdehyde; SOD: superoxide dismutase; CAT: catalase; Nrf2: nuclear factor erythroid-derived 2-like 2; MCP-1: monocyte chemoattractant protein-1; BMP-2: bone morphogenic protein-2; BUN: blood urea nitrogen; HAP: hydroxyapatite. ↑ = increases and ↓ = decreases.

## Data Availability

No new data were created or analyzed in this study. Data sharing is not applicable to this article.
